# Supplementary material for: HIV treatment response among female sex workers participating in a treatment as prevention demonstration project in Cotonou, Benin
Source: PLoS One. 2020 Jan 23;15(1):e0227184. doi: 10.1371/journal.pone.0227184 (PMC6977752; doi:10.1371/journal.pone.0227184)
Supplement: Attachment — Submitted filename: Response to reviewers.docx [file pone.0227184.s002.docx]

September 15^th^, 2019

Dr Orna Mor, Ph.D.
Academic Editor
PLoS ONE

**RE: Response to reviewers - Manuscript ID: PONE-D-19-15725**

Dear Editor,

We would like to sincerely thank the editor and the reviewers for the comprehensive evaluation of our manuscript entitled “***HIV treatment response among female sex workers participating in a treatment as prevention demonstration project in Cotonou, Benin***” that has been submitted for publication in PLOS ONE.

We are excited the reviewers found our manuscript to be of significance to the field. Their pertinent comments and suggestions have definitely contributed to strengthen our paper, and as such, we have done our best to address all the editor’s and reviewers’ concerns. These changes are highlighted in the manuscript with the Word “*Track changes*” function. Since major revisions were brought and as requested in the letter from the academic editor, a “clean” version of the manuscript is also provided. As the revised manuscript also includes two figures, we have uploaded the figure files separately and have written the figure title and legends following the text that explains about the figure.

Please find below the comments and questions (numbered and italicized) followed by our answers and details about the corrections that we performed on the original manuscript. Please note that when we refer to line numbers, those refer to line numbers as read in the clean version of our revised paper without tracked changes.

The revised manuscript is now ready for further consideration by *PLoS ONE*. We sincerely hope that it will now be suitable for publication in your very interesting journal.

Best Regards,

Mamadou Diallo and Michel Alary (on the behalf of all coauthors)

***Note from the Editor:***

*1) We note that you have indicated that data from this study are available upon request.*

**Response:** Indeed, as we mentioned while submitting the manuscript, data are available and can be made accessible. We have now provided a minimal anonymized data set necessary to replicate our study findings. This file uploaded as a supporting Information file can be accessed in the PloS ONE website.

***Additional Editor Comments (if provided)***:
*The authors report on the results of a TasP demonstration study among a small cohort of 107 HIV-infected, treatment naïve, female sex workers in Cotonou, Benin. In general, the study is clear and well written and interesting. However, the number of tables versus size of study is uncomparebly high.*

*2) Table 1 could be removed and explained in the text*

**Response**: We have removed the table 1 and explained the corresponding data in the text (page 12-13: line 230-233).

*3) A graph of cd4 and vl versus visits could replace tables 3 and 4.*

**Response**: We have replaced tables 3 and 4 by Figures 1 and 2 (2 figures needed to provide enough details) that we have uploaded as separate files. Figure titles and legends are provided in the manuscript itself (see page 19, lines 282-289 for changes in CD4, and page20-21: lines 313-318 for viral response). We have also slightly modified the text describing these 2 figures to provide some of the actual numbers that were in the table and that are somewhat less clear in a figure format (see page 18-19, lines 269-276 for changes in CD4 and page 20, lines 300-308 for viral load).

*4) Table 5 is unclearly presented*

**Response:** We have entirely reformatted table 5, which is now table 2 (see page 21).

*5) On the other hand, a table summarizing the prevalence and type of baseline DRM in the different class of drugs versus subtypes is missing.*

**Response**: The information on baseline DRM was already present in former Table 6; indeed, we agree it was badly formatted. We have also entirely reformatted this table that is now Table 3 (see page 24-25). In addition, we now provide data on baseline prevalence of DRM in the text (page 23, lines 350-367).

*6) The outline of Table 6 is also unclear and if a new summary table is provided- then table 6 can be in appendix.*

**Response**: See our response to the previous comment.

*7) Please also check if all references are correctly written and according to PLOS policy.*

**Response**: We have checked the references and adjusted them to PLOS policy where necessary.

***Reviewer #1: General comments:***

8) *The authors report on the results of a Treatment as Prevention demonstration study among a cohort of 107 HIV-infected, treatment naïve, female sex workers in Cotonou, Benin. Their findings on acceptability, retention and viral load provide important and relevant information which should be taken into account when implementing TasP among key populations in Africa. The general methods seem adequate. Statistical methods and analysis however should be verified. Some of the statistical tests used are not mentioned. The authors use generalized estimating equations to analyse time trends and the association between adherence and virological response however the results of the model are not clear and complete. It is also a pity that no attempt for identifying or adjusting for confounders have been made.*

 **Response**: We thank the reviewer for addressing this aspect of the methodology of data analysis, and his willing to have more information in the methodology. We hereby provide detail information on the methodology and reasons we used for the analysis. Indeed, since we had repeated data, the use of generalized estimating equations with the log –link and the type3 test of Wald seemed to be more appropriate for the analysis of time trend changes as well as the association between viral load and self-reported adherence. While analyzing the association between viral load and self-reported adherence, the final model controlled for time duration since ART initiation. We also initially included potential confounders such as age, country of origin, duration in sex work, having a regular partner (or not), and number of clients (but not reported condom use as it was reported to be extremely high: see Table) in the multivariate model, however, all these variables did not confound the association between self-reported adherence and viral load. Therefore, we did not keep them in the final model in order to maximize statistical power. We have made some changes in the section on statistical methodology to better describe our approach (see page 11, lines 197-202) as well as in the results section when reporting the associations between self-reported adherence and viral load (see page 21, lines 324-332).

Specific comments:

**Introduction**
*9) - P5, line 59: “an estimated 6.1 million people living with HIV at the end of 2016”
Please update the numbers and add a reference.*

**Response:** We have updated both the numbers and the reference. (Page 5: line 58-60).

*10) - P5, line 73: “(12,13).(14)”
Please correct this typo.*

**Response:** Corrected (page 5: line 73).

*11) Methods*
- P.4, line 93: “FSW not known to be HIV-positive on ART”
This expression is not clear, why the specification “on ART”? Do you mean “FSW not known to be HIV-positive”?

**Response:** We thank the reviewer for pointing out this ambiguity. Indeed, this expression was misplaced. It’s only after the confirmation of the screening test at the Dispensaire IST, that we restrained recruitment to HIV-positive FSWs who were not known to be on ART. Being ART naïve were confirmed following responses to questions, and confirmation the clinic records as the vast majority of HIV-positive FSWs in Cotonou who are on ART receive their treatment at Dispensaire IST). In the manuscript, the deletion at the inappropriate location of the manuscript is on page 6: line 90-91 and the addition is on page 6, lines 93-95.

IST (in order to confirm that participants were ART-naïve)

*12) - P. 8, lines 130-132. Syphilis tests.
There may be some confusion when using the terms “rapid test” and “RPR”. Can you make it clear that the rapid is a treponemal, and the RPR is a nontreponemal test. One could also argue that one positive RPR test is not sufficient to detect active syphilis. According international guidelines there is a need of a fourfold change in titer, equivalent to a change of two dilutions to demonstrate an active infection.*

**Response:** We have now specified that the rapid test was treponemal and that the RPR was non-treponemal (page 8: Line 130-133). Note also than in cross-sectional studies (and all data presented in table 1 are from baseline and thus cross-sectional), it is usual to apply the definition we used for active syphilis. We don not report follow-up data on this issue, but please note that, in addition to not having a single case of active syphilis at baseline, there was no new case of syphilis during follow-up.

***Results*** *13) - P. 13, line 237: “13.2 ± 7.7”
Please specify that 7.7 is a standard deviation (I guess)*

**Response:** This is now specified (page 12: line 230).

*14) - P. 13, line 241: “the main reasons for not completing final visits”
The reason for not completing final visits is not the main issue I guess, but the reason for not being retained on ART, whether they come for a final visit or not.*

**Response:** In fact, we cannot know if the women who did not complete follow-up in the study were retained on ART or not. It depends if they accessed ARV clinics after leaving the study (most of them were not in Cotonou anymore and many were in other countries). Therefore, the reasons given in the manuscript are for withdrawing from the study. The wording of this section has also been changed because of the deletion of original table 1 and the addition of new text following a request from the editor (see page 9: 230-236).

*15) - P. 15, line 277: “were infected or had a history of NG/CT”
Was this a history of an infection “ever”? It would make more sense to separate the biological results (STI at baseline) and the interview data “did you ever had an STI”, because they are two separate things.*

**Response:** We thank the reviewer for pointing out this mistake. The prevalence we provided was in fact for the actual biological results of the nucleic acid amplification tests for NG and CT and did not include past history collected by questionnaire. This is now corrected (see page 13, lines 249-252).

*16) - P .20, table 3 “Mean”
Please add “Mean CD4” for clarity in the title and the column head*

**Response:** As per the editor’s request, we have replaced table 3 by figure 1, but mean CD4 count is now specified in this figure’s title and legend (page 19: Line 282-292).

*17) - P. 20, table 3: p-values
The statistical methods (tests) for this table are not shown, please indicate which tests were used to obtain both type of p-values (p-trend and p-value), and explain in the methods.*

**Response:** We described the analysis methodology in the method section. For all the analysis, we used generalized estimating equations with the log–link and the type3 test of Wald (see page 11: Lines 197-202), (see also our response to comment 8 above). Note that table 3 has now been replaced by figure 1.

*18) - P. 23, table 4: p-values
Idem as previous comment.*

**Response**: Same response as to the previous comment. Note also that table 4 has now been replaced by figure 2. (see page 11: Lines 197-202).

*19) - P. 24, lines 363-367: “the proportion … increased with increasing adherence (p=0.06 and 0.003)”. The results in the table do not confirm this statement: the proportion of visits where viral load was suppressed was 83.2% for the group with 90% adherence and 84.2% for the group 75-89% adherent.*

**Response**: We used 4 adherence categories in this analysis and evaluated a test for trend over the 4 categories and these are the p-values we obtained (see our response to comment 8 above). This can happen even if the various categories yield similar prevalence ratios when compared to the reference category as long as the association is strong enough. However, we agree with the reviewer in the fact that it would be important to qualify this statement by evoking the stability of the prevalence ratios as long as the adherence level is >50%. We have thus added a sentence in this regard in the result section (see page 21, lines 330-332).

*20) - P. 24, table 5: results GEE
The table is quite difficult to read. The column with the head “Ratio” seems to present prevalences, not prevalence ratios. A prevalence is also a ratio in theory, but in this situation the term “ratio” adds to confusion.*

**Response:** As per the editor’s request, we have completely reformatted this table, that is now table 2, and we have changed the column header as suggested by the reviewer. (see page 21-22: line 334-340).

*21) - P. 24, table 5, p for trend (p=0.06 and 0.003)
Which test has led to these p’s? It is curious to see a p for trend of 0.06, when the PR are exactly the same in all adherence categories (with the exception of the reference category). Idem for the p for trend 0.03 for the outcome “undetectable”, where PR go from 3.2 for the group with the best adherence, vs 3.3 for the group with adherence 75% and 50%* (page 15: line 334).

**Response:** See our response to comments 8, 17 and 19 above.

*22) - P. 24, table 5: results GEE
The table only show the PR of the virological response per adherence level, the time factor is not presented. Have the authors considered to check potential confounders including age, behaviour factors such as condom use? This may provide interesting alternative explanations for the relation adherence/virological outcome.*

**Response:** As time since treatment initiation was treated as a confounding factor for the association between adherence levels and viral load and that we were mainly interested by this latter association, we do not present prevalence ratios for this time variable to keep the table as simple as possible for the readers. Concerning the other potential confounders, see our response to comment 8 above.

*23) - P. 28-30, table 6
Please revise lay-out of the table, as the text in the lines is not positioned in a standardized way, which make the table difficult to read.*

**Response:** This table has now been entirely reformatted and its number as changed to table 3 (see page 24-26).

**Discussion**

*24) - P. 31, line 440-441
Revise structure. The logical flow can be improved by replacing: “since half of them are not Beninese, and even when they were …”*

**Response:** We have reviewed and rephrased the sentence as suggested *(page28: line 418-423).*

*25) - P. 32, line 453: “mobility”
Mobility is indeed be a serious barrier. The authors may discuss here the access of the FSW to ART services if they move to another place, another country.*

**Response:** We added some discussion on this issue (see page 29, lines 433– 443).

*26) - P. 32, line 466
Correct typo: “fact”*

**Response:** Corrected (page 29: line 433)

*27) - P. 34, lines 509-512: “The results revealed … interventions”
This paragraph is repeated later in the discussion. It is a general conclusion and should be moved to the end of the paper.*

**Response:** We removed this paragraph as suggested by the reviewer (see page 32, lines 499-502)*.* At the same time, since this sub-section now only present limitations, we changed the name of the sub-section “Study strengths and caveats” to “Study limitations”.

*28) - P. 35, lines 527-529
The authors should be cautious in their conclusions about the association between self-reported adherence and viral load, taking into account the results (see higher)*

**Response**: We have made some changes in order to be more cautious with this conclusion (see page 32, line 506-510).

***Reviewer #2****: General comments*

*29) Although the study describes a TasP/PrEP demonstration project, limited data are provided about PrEP*

**Response**: We thank the reviewer for his interest in the PrEP data. Although our project focused on TasP and PrEP, we only report data on TasP in this paper. Indeed, we have already published a paper on the overall resust of our TasP/PrEP study in the Journal of the International AIDS Society (JIAS) (see 20 of the present study or DOI: [10.1002/jia2.25208](https://doi.org/10.1002/jia2.25208)). The title of this paper is “*Early antiretroviral therapy and daily pre-exposure prophylaxis for HIV prevention among female sex workers in Cotonou, Benin: a prospective observational demonstration study*. However, the results presented in the present paper include some additional TasP subjects and present analyses that are not included in our JIAS paper.

*30) The accession numbers of the nucleotide sequences generated in this project are not provided*

**Response**: We perfectly agree with the reviewer that the nucleotide sequences generated in this project should be provided. Unfortunately, we did not think about recording these sequences in Genbank. However, if requested, we can provide s the sequences for verification.

*31) The number of Tables should be reduced*

**Response**: This has been done: we went from 6 tables to 3 tables and 2 figures following the editor’s comments.

*32) Page 26, HIV strains and drug resistance (details in Table 6)*

*Authors provide subtype classification twice (i.e. CRF02_AG/CRF02_AG). This should be explained*

**Response:** As to our understanding of the question, the reviewer asks us to explain why we reported the same subtype twice (i.e. CRF02_AG/CRF02_AG). This is a standard terminology for presenting the results of genotyping for drug resistance. Indeed, sequencing the HIV-1 *pol* and *env* genes is routinely used to identify mutations associated with resistance to reverse transcriptase (RT) or protease (PR) inhibitors in HIV-1 circulating recombinant forms (CRFs) including CFR02_AG and CRF01_AE. Samples were genotyped at both env and pol regions. When the results yielded identical sequences in the two regions, we reported the same sequence twice (ex.: CRF02_AG/CRF02_AG), otherwise we reported differently (i.e. CRF11-cpx/CRF02_AG). This appears in the revised table 3 (i.e. G/CRF02_AG and CRF11-cpx/CRF02_AG) and shows the different strains in our study.

*The prevalence of resistance mutations should be reported*

**Response:** We now report the prevalence of resistance mutations in the text of the manuscript (see page 22-23, lines 345-353).
