# Supplementary material for: HIV treatment response among female sex workers participating in a treatment as prevention demonstration project in Cotonou, Benin
Source: PLoS One. 2020 Jan 23;15(1):e0227184. doi: 10.1371/journal.pone.0227184 (PMC6977752; doi:10.1371/journal.pone.0227184)
Supplement: Attachment — Submitted filename: Response to reviewers_ii.docx [file pone.0227184.s003.docx]

December 5^th^, 2019

Dr Orna Mor, Ph.D.
Academic Editor
PLoS ONE

**RE: Response to reviewers - Manuscript ID: PONE-D-19-15725R1**

Dear Editor,

We would like to sincerely thank both the editor and reviewers for the comprehensive evaluation of our manuscript entitled “***HIV treatment response among female sex workers participating in a treatment as prevention demonstration project in Cotonou, Benin***” that has been submitted for publication in PLOS ONE.

We are more than excited that the previous corrections were made to the reviewers’ satisfaction, and that there remains only few minor remarks to address on the revised version. We are confident that these pertinent comments and suggestions will definitely contribute to strengthen our paper, and make it clear for the audience. As such, we have done our best to address all the editor’s concerns.

These changes are highlighted in the manuscript with the Word “*Track changes*” function. Since the revisions are relatively substantial and as requested in the letter from the academic editor, a “clean” version of the manuscript is also provided.

Please find below the comments and questions (numbered and italicized) followed by our answers and details about the corrections that we performed on the original manuscript. While addressing the raised questions, the endnote software induced some changes in the references citations; we have also addressed these changes. Please note that when we refer to line numbers, those refer to line numbers as read in the clean version of our revised paper (manuscript) without tracked changes.

The revised manuscript is now ready for further consideration by *PLoS ONE*. We sincerely hope that it will now be suitable for publication in your very interesting journal.

Best Regards,

Mamadou Diallo and Michel Alary (on the behalf of all coauthors)

***Note from the Editor:***

*1) Page 8 line 139: Neisseria gonorrhoeae (NG) and Chlamydia trachomatis (CT). In Table 1: N. gonorrhoeae or C. trachomatis. Please choose either NG/CT or N. gonorrhoeae - C. trachomatis throughout the text.*

**Response:** We have chosen to use *N. gonorrhoeae* and *C. trachomatis* throughout the text (page 8, line 140-141; page 14 line 257; table 1); except for the name of the Probetec assay used to test for these infections, as NG/CT is part of the brand name of the assay.

*2) Page 9 148-151: nuclisens can officially assess HIV-1 only (not HIV-2). Only one publication (ref 21) assessed its performance on HIV-2 subtype A samples only and claims detection limit of 200 c/ml. This is not what is stated here. I suggest to remove HIV-2 from this sentence.*

**Response**: Indeed, Biomerieux-diagnostics mentioned that NucliSens^®^ HIV-1 QT assay is generally insensitive for detection of HIV-2 RNA. In addition, samples from individuals infected with HIV-2 may exhibit cross-reactivity in this assay. We therefore, have removed HIV-2 from this sentence, as suggested (page 9: line 151). Please also note that, as already stated in the manuscript, subjects with evidence of HIV-2 infection were excluded from the study.

*3) Also the limit of quantitation for HIV-1 has changed during the years. Please provide the version number of the VL kit used in this study and give the updated LOQ for the version used herein (as far as I recall it was never 40c/ml).*

**Response**: We used the NucliSens EasyQ® HIV-1 v2.0 kit. 48 tests - Ref: 28 5033. The kit is of high-level sensitivity with validated input volumes plasma, can detect the majority of HIV-1 subtypes including: A, B, C, D, F, G, H, J, CRF01_AE and CRF02_AG with a viral load measuring range between: 10-10 000 000 copies/ml.

This kit provides detection limits according to volume of plasma used:

| **Flexible Input** | **Limit of Detection (LoD)** |
| --- | --- |
| 1 ml plasma | 25 cps/ml |
| 0.5ml plasma | 50 cps/ml |
| 0.1 ml plasma | 292 cps/ml |
| Dry Blood Spot (0.1 ml) | 802 cps/ml |

We used 0.5 ml plasma, therefore the lowest viral load we could amplify was 50 copies/ml (page 9: line 151).

*4) Page 9 line 151: Replace HIV by HIV-1 (to exclude HIV-2)*

**Response:** We have replaced HIV by HIV-1 (page 9, line 152).

5) Page 10 line 179: vl <40 cannot considered undetectable. If 40 is the quantification limit it could be considered as below quantification limit. If <40 is the LOQ for the assay used herein, please amend the term “undetectable” (in cases of VL <40 C/ML) to “below quantification limit” all through this paper.

**Response:** Thank you very much for underlining this ambiguous misuse of “*undetectable*” instead of “below *quantification limit*”, “undetectable” being a term too often used in viral quantification. Since we used the NucliSens EasyQ® HIV-1 v2.0 kit. 48 tests - Ref: 28 5033 and 0.5 ml plasma, the lowest viral load we could amplify was 50 copies/ml. *We therefore have* amended the term “undetectable” (in all cases of VL <40 C/mL) to “below quantification limit” all through the manuscript, and the figure 2. Please, see (page 3: line 43-45; page 9: line 151; page 10 line 178-185; page 11 line 201-202; page 18 line 304-307; page 19 line 313-327; page 20 line 338-340, table 2; page 26 line 397).

*6) Page 18 line 282: Fi 1 change to Figure 1*

**Response:** We have changed *Fi 1 to Figure 1* (page 18: line 290).

*7) Page 19* line 296: term “suppressed viral load” and “undetectable viral load” should be explained in the text and not only in the figure legend, especially as <1000 is not regularly considered “suppressed…” Please discuss to these numbers in the discussion section as today VL >200 (SOMETIMES >50) is considered virological failure.

**Response**: The term viral suppression or “*suppressed viral load*”, when viral load is <1000 copies/mL was used to evaluate treatment success by the World Health Organisation (WHO) and the Joint United Nations Programme on HIV/AIDS (UNAIDS). According to the 2016 WHO Consolidated guidelines on the use of antiretroviral drugs for treating and preventing HIV infection, a viral load <1000 copies/mL defines treatment success. The term “undetectable” viral load was defined as viral load below detection limits of the assay used for viral load quantification in the study (page 10 line 180-184, page 26 line 400-402). For the latter, we however now use “below quantification limit” instead of “undetectable” throughout the manuscript.

*8) Table 1: Needs restructuring. Numbers are not in lanes and some do not fit the space*

**Response**: We have restructured Table 1 so that numbers are in lanes and fit the space (page 14-17).

*9) Table 3: needed reformatting and detailing. What does NA mean? Why some spaces are empty? The list of ART for each sample is the drugs which could be taken and are no affected by the identified DRM? Why to include drugs that are not in use any more? The subtype is given for both RT and PR? Please provide detailed information so that the reader will easily understand.*

**Response table 3**: NA meant not applicable as there was no resistance mutation to this class of ARVs; some spaces were empty for the same reason. We have now decided to leave all these spaces empty when no resistance mutation was present to a given ARV class. The list of specific drugs is quite useless at the end and we have suppressed this column entirely in order to make the table easier to read. We have provided the sequences corresponding to the protease and the reverse transcriptase regions (PR/RT), and this is now better explained (table page 23-24: table 3).
